# Short-Term Outcomes and Predictors of Effectiveness of Tocilizumab in Systemic Juvenile Idiopathic Arthritis: A Prospective Cohort Study

**DOI:** 10.3389/fmed.2021.665028

**Published:** 2021-05-10

**Authors:** Doaa W. Nada, Abdelkawy Moghazy, Abdallah El-Sayed Allam, Alessia Alunno, Amira M. Ibrahim

**Affiliations:** ^1^Physical Medicine, Rheumatology and Rehabilitation Department, Faculty of Medicine, Tanta University, Tanta, Egypt; ^2^Rheumatology and Rehabilitation Department, Faculty of Medicine, Cairo University, Cairo, Egypt; ^3^Morphological Madrid Research Center (MoMaRc), Madrid, Spain; ^4^Rheumatology Unit, Department of Medicine, University of Perugia, Perugia, Italy; ^5^Physical Medicine, Rheumatology and Rehabilitation Department, Faculty of medicine, Kafr El Sheikh University, Kafr el-Sheikh, Egypt

**Keywords:** systemic juvenile idiopathic arthritis, tocilizumab, inflammation, c reactive protein, erythrocyte sedimentation rate, ferritin

## Abstract

**Background:** Systemic Juvenile Idiopathic Arthritis (sJIA) is a unique category of juvenile arthritis in which interleukin 6 plays a major pathogenic role. This study aimed to describe the therapeutic short-term outcomes among patients with sJIA starting tocilizumab (TCZ) therapy and to identify possible predictors of treatment response.

**Methods:** We conducted a prospective observational study including 65 patients with sJIA meeting ILAR classification criteria with active disease despite conventional therapy that were treated by TCZ between August 2019 and October 2020 as the first-line biological therapy. Clinical and serological parameters were recorded at baseline and after 1 year of TCZ therapy.

**Results:** After 1 year, 25% of the patients achieved minimal disease activity and 35% achieved clinically inactive disease. A significant reduction of the 10-joint juvenile arthritis disease activity score and acute phase reactants was also observed. Patients with younger age (≤7 years), shorter disease duration (≤3 years), lower disease activity, and higher serum ferritin and systemic manifestations showed more favorable results.

**Conclusion:** Patients with sJIA showed favorable disease outcomes with TCZ treatment for 1 year, especially if the drugs were administered earlier in the disease course and in younger patients with a more pronounced inflammatory status. Our results may help to define the profile of patients with sJIA who are more likely to benefit from IL-6 blockade.

## Introduction

The term juvenile idiopathic arthritis (JIA) encompasses all forms of arthritis that occur before 16 years of age and persists for at least 6 weeks with other causes of arthritis being ruled out. It occurs in a frequency of 3 in 10,000 among children (1–5) and according to the ILAR classification it is classified into seven variants (6, 7). Systemic JIA (sJIA) represents a clinically distinct category that accounts for 10–20% of JIA patients with no gender predominance; systemic involvement including fever, rash, and enlarged lymph nodes; hepatosplenomegaly; and serositis (8). Genetic studies have shown marked variation in the loci associated with sJIA compared to other ILAR categories (7). In addition, a marked activation of the innate immune system was demonstrated and specific pro-inflammatory cytokines (e.g., IL-1 and IL-6) contribute to the multisystem inflammation and may ultimately lead to 5–8% of patients having the most severe complication of sJIA, the macrophage activation syndrome (MAS) (8). On this basis, the treatment approach of sJIA has been shifted from the use of TNF inhibitors (TNFi) to that of IL-6 inhibitors [such as tocilizumab (TCZ)] and IL-1 blockers (such as Anakinra or Canakinumab) (9–13). The 2015 National Health Service England treatment pathway, American College of Rheumatology/Arthritis Foundation (ACR), as well as Childhood Arthritis and Rheumatology Research Alliance Consensus (CARRA) recommended that patients with sJIA should be prescribed TCZ or Anakinra as a first biologic therapy following failure of conventional therapy (14–16). TCZ, a humanized monoclonal antibody directed against IL 6 receptor, is the first biological drug approved in Japan for treatment of sJIA alone or with methotrexate (MTX) (17–21). A number of possible predictors of the therapeutic effectiveness of IL-1 inhibitors have been reported, and these include less severe joint disease, increased neutrophil or white blood cell count, shorter disease duration, older age at disease onset, and use of IL-1 blockade as first-line therapy, particularly in patients with new-onset disease and not yet exposed to steroid or disease-modifying anti-rheumatic drugs (DMARDs) (21–27). However, the experience gained on IL-6 inhibitors in sJIA is still limited; hence, there is a need to better characterize the profile of patients with sJIA who are more likely to respond to IL-6 blockade. This study aimed to describe the therapeutic short-term outcomes among patients with sJIA treated with TCZ and to investigate the possible therapeutic predictors of treatment response.

## Materials and Methods

### Study Design and Population

We conducted a prospective observational study including patients with sJIA according to the ILAR classification criteria (2), who were starting TCZ in Tanta & Kafr El Sheik Health Insurance Hospitals, as well as Outpatient & inpatient clinics of Physical Medicine, Rheumatology & Rehabilitation Department, Tanta University Hospitals, between August 2019 and October 2020. Patients with other rheumatic or autoinflammatory diseases were excluded. A previous or concomitant treatment was allowed for NSAIDs, systemic or intra-articular glucocorticoids, and conventional synthetic (cs) DMARDs (e.g., MTX). Corticosteroids were standardized for all patients and given at a median dose of 0.5 (0.2–1) mg/kg/day. Patients were excluded if they were in minimal disease activity (MDA) at the start of TCZ with no systemic features present or if received any biologic (b) DMARDs before the start of the study. TCZ was given at a dose of 8–12 mg/kg slow intravenous infusion every 2 weeks. At baseline and after 1 year, a detailed questionnaire on patient demographics, clinical and laboratory disease characteristics, as well as all current and past DMARDs and changes in the therapeutic strategy were collected. Quantitative measurement of the activity of systemic disease was performed using a systemic manifestation score (SMS) (Table 1) devised by weighting extra-articular features (27). It was decided to assign a greater weight to fever owing to its greater effect on a child's well-being and major importance in driving treatment decisions. The SMS ranges from 0 to 10, (where 0 = absence of systemic manifestations) and 10 = maximum activity of systemic manifestations). The study was conducted in accordance with the declaration of Helsinki (28) and written informed consent was obtained by the parents or, if not possible, by the next of kin. We followed the recommendations of STROBE guidelines during the preparation of this manuscript (29).

**Table 1:** Systemic manifestation score.

Fever = 37–38°C	1 point
Fever = 38–39°C	2 point
Fever = 39–40°C	3 point
Fever >40°C	4 point
Rash	1 point
Generalized lymphadenopathy	1 point
Hepatomegaly and/or splenomegaly	1 point
Serositis	1 point
Anemia (hemoglobin <9 g/dl)	1 point
Platelet count > × 10^9^/L or ferritin >500 ng/ml	1 point

Baseline disease characteristics were assessed, including the 10-joint juvenile arthritis disease activity score (JADAS-10) that ranges from 0 to 40. It is a composite score resulting from the addition of four values: physician's global assessment (PGA) on a 0–10 visual analog scale (VAS) (0 = no activity; 10 = maximum activity); patient (or parent) global evaluation of well-being (PGE) on a 0–10 VAS scale (0 = no activity; 10 = maximum activity); active joint count (AJC), up to 10 joints, irrespective of their type, censored at 10; and normalized ESR (value in mm/h −20)/10, where values 20 mm/h are converted to 0 and values of more than 120 mm/h are converted to 120 (30).

### Study Outcomes

Primary outcome measures were investigated at 1 year after start of biologic therapy and included proportion of patients achieving MDA or clinically inactive disease (CID). Patients with sJIA were defined as achieving MDA if the PGA was not >3.4 cm, the PGE was not >2.1 cm, with a maximum of one active joint (31). Patients were defined as achieving CID if they had no active joints, no systemic features, no active uveitis, and a PGA of zero (9, 32). Secondary efficacy outcomes included the change in active joint count, PGA, PGE, JADAS-10, systemic disease score, laboratory results, and drug doses. A complete clinical response (CCR) was defined according to the following five criteria, which should all be present: (1) absence of fever; (2) PGA of the overall disease activity ≤ 1; (3) count of active joints ≤ 1; (4) negative CRP; and (5) ≥75% reduction of dosage of corticosteroid therapy from baseline. In line with the intention-to-treat principle, patients who discontinued TCZ before reaching 1 year or had treatment for any reasons other than disease remission were classified as non-responders. Patients who discontinued TCZ before 1 year because of disease remission and had remained in such a state until 1 year were considered responders (33).

### Statistical Analysis

The statistical package used was IBM-SPSS version 21. Descriptive statistics are presented as medians and interquartile range (IQR) for continuous variables, and mean and standard deviation for discrete variables, and number and percentages for categorical variables. Comparisons of quantitative variables between responder and non-responder groups were made by Mann–Whitney *U* test. Multivariable logistic regression analysis was performed, entering baseline variables that showed significant results in univariate tests (*p* < 0.05) or were considered to be of foremost importance for the study outcome, with CID as the outcome variable. Before the application of logistic regression procedures, some quantitative variables were dichotomized to binary variables, using the cut points obtained through the receiver operating characteristic (ROC) curve analysis. Variables that were significantly associated with the study outcomes were identified and expressed as odds ratio (OR) and 95% confidence interval (CI); statistical significance was tested by likelihood ratio test; *p*-values <0.05 were considered significant. The area under the ROC curve of the best-fitting model was used as an indicator of the predictive ability of the model.

## Results

A total of 65 sJIA patients (44 females and 21 males) with a median age at baseline of 6.5 years receiving TCZ as first-line bDMARD were enrolled in the study. Five patients (7.7%) withdrew from the study due to either hypersensitivity at the first TCZ administration (*N* = 2) or loss to follow-up (*N* = 3). The baseline demographic, clinical, and laboratory features of the 60 patients completing the study also divided according to response to TCZ are presented in Table 2.

**Table 2:** Baseline demographic and clinical data of all systemic JIA patients including the responders and non-responders to tocilizumab therapy.

	**All patients (*n* = 60)**	**Non-responders (*n* = 24)**	**Responders (*n* = 36)**	***p*-value**
Age, years, Median (IQR)	6.5 (2–15)	7.25 (2–15)	6.0 (2–13)	0.327
Age at onset, years, Median, (IQR)	3.75 (1–13)	5.5 (1–13)	3.0 (1–11)	0.047
Disease duration, years, Median (IQR)	2.5 (1–7)	5.0 (1–7)	2.0 (1–5)	<0.001
Corticosteroid dose (mg/kg/day), Median (IQR)	0.5 (0.2–1)	0.9 (0.5–1)	0.5 (0.2–1)	0.010
Systemic manifestations score, Median (IQR)	3.5 (2–5)	2.0 (2–4)	4.0 (3–5)	<0.001
AJC, Median (IQR)	6.5 (4–9)	7.0 (6–8)	5.5 (4–7)	0.053
JADAS10, Median (IQR)	26.65 (18.5–36)	26.4 (18.5–36)	23.8 (18.5–32.7)	0.006
PGA, Median (IQR)	7.0 (3–10)	8.0 (4–10)	8.6 (2.5–10)	0.022
PGE, Median (IQR)	7.5 (4–9)	7.0 (4–9)	7.5 (5–9)	0.795
ESR (mm/h)	8.0 (2.5–10)	5.6 (2.5–10)	8.6 (2.5–10)	0.027
CRP (mg/dl), Median (IQR)	54.85 (14.5–112)	46 (24–87)	54.85 (14.5–112)	0.399
HB (<9 g/dl), Median (IQR)	9.60 (8.2–10.8)	9.2 (8.2–10.1)	9.7 (8.3–10.8)	0.047
WBC (count × 10^9^/L), Median (IQR)	7.70 (3.5–12.2)	5.6 (3.5–8.9)	8.5 (5.1–12.2)	<0.001
Platelet count (>600 × 10^9^/L), Median (IQR)	487 (323–975)	367 (323–730)	520 (365–975)	<0.001
Serum ferritin (ng/ml), Median (IQR)	400 (160–480)	156 (44–570)	1,257.5 (200–2,000)	<0.001

*Data are expressed as median (IQR)*.

*p-value indicates the comparison between non-responders and responders. AJC, active joint count; PGA, physician global assessment of overall disease activity; PGE, patient (parent) assessment of overall well-being; JADAS10, 10-joint juvenile arthritis disease activity score; ESR, erythrocyte sedimentation rate; CRP, C-reactive protein; Hb, hemoglobin; WBC, white blood cell; Systemic manifestation score (0–10 points)*.

At 1 year after the start of tocilizumab, 25% of the patients achieved MDA and 35% achieved CID. Corresponding unadjusted and propensity adjusted OR (95% CI) are shown in Table 3.

**Table 3:** Primary outcomes after 1 year of Tocilizumab therapy.

	***N* (%)**	**Unadjusted OR (95% CI)**	**Propensity adjusted OR (95% CI)**
Minimal disease activity	15 (25%)	1.20 (0.45–3.91)	1.20 (0.35–4.41)
Clinically inactive disease	21 (35%)	2.6 (0.84–8.3)	2.7 (0.64–11.3)
Active disease	24 (40%)	2.1 (0.57–7.6)	1.8 (0.47–7.7)

*Propensity score included gender, age, disease duration, concomitant methotrexate, concomitant steroids, active joint count, physician global assessment of overall disease activity (PGA), patient (parent) assessment of overall well-being (PGE), ESR, and JADAS-10: 10-joint juvenile arthritis disease activity score*.

Notably, 35% of the included patients had a reduction of their TCZ dose, 30% remained on the same dose, while 35% increased their dose at the end of 1-year follow-up. In addition, 25% stopped corticosteroid concomitant treatment and the remaining 75% reduced the dose along the course of treatment. The median (IQR) dose of prednisone at the beginning of treatment was 0.9 (0.5–1.0) mg/kg/day and 0.5 (0.2–1.0) mg/kg/day in non-responders and responders, respectively, and at 1 year it was 0 (0–0.16) mg/kg/day and 0 (0–0.14) mg/kg/day and in non-responders and responders, respectively. Regarding csDMARDs, of the 20 patients receiving MTX at baseline, 45% of patients stopped therapy by 1 year of follow-up. Moreover, 27 (45%) of our patients met the criteria for CCR at 1 year after the start of TCZ, whereas 33 patients (55%) did not. Of the 33 patients who were non-responders, 15 had the medication withdrawn before the study endpoint for inefficacy (7 patients), adverse events or intolerance (5 patients), and difficulty in the delivery of the drug by the local pharmacy (3 patients). The two latter patients were kept in the analysis as non-responders in line with the intention-to-treat principle. The median time to withdrawal was 0.79 months (IQR 0.39–1.35). Of the 27 patients who had CCR at 1 year, 20 (74%) were still receiving TCZ but had discontinued all other medications, the median time to withdrawal of corticosteroids was 0.93 months (IQR 0.55–1.77) and 7 patients (26%) were still taking low-dose corticosteroids and/or synthetic DMARD in combination with TCZ. Table 4 shows the change from baseline and *p*-values of the secondary outcome variables.

**Table 4:** Secondary outcomes absolute values and change from baseline after 1 year of tocilizumab therapy.

**Variable**	**Absolute value, mean (SE)**	**Change from baseline, mean (SE)**	***p*-value**
PGA, (0–10 cm VAS)	1.23 (0.15)	−5.75 (0.11)	<0.001
PGE, (0–10 cm VAS)	1.71 (0.15)	−5.54 (0.02)	<0.001
AJC	0.76 (0.09)	−6.1 (0.04)	<0.001
JADAS 10	3.84 (0.47)	−23.34 (0.11)	<0.001
ESR (normalized) (mm/h)	0.51 (0.11)	−6.32 (0.24)	<0.001
CRP (mg/dl)	6.30 (0.75)	−49.79 (2.8)	<0.001
Serum ferritin (ng/ml)	112.70 (8.76)	−604.18 (65.84)	<0.001
Hb (g/dl)	10.83 (0.12)	1.41	0.63
Platelet count (n/mmc)	285.61 (12.95)	241.21 (12.85)	<0.001

*AJC, active joint count; PGA, physician global assessment of overall disease activity; PGE, patient (parent) assessment of overall well-being; JADAS10, 10-joint juvenile arthritis disease activity score; ESR, erythrocyte sedimentation rate; CRP, C-reactive protein; Hb, hemoglobin*.

Multivariable logistic regression analysis of the treatment response showed that younger age at disease onset (≤5 years), lower disease duration (≤3 years), and lower disease activity with higher serum ferritin and systemic manifestations were associated with more favorable response (Table 5).

**Table 5:** Logistic regression of predictors for effective response to tocilizumab therapy.

**Variables**	**OR**	**95% CI**	**AUC**	*p*-value^*^
Age at onset ≤5 years	2.11	1.53–27.40	0.47	0.002
Disease duration ≤3 years	6.50	1.28–34.21	0.46	0.003
JADAS10 ≤10	8.10	1.27–47.10	0.53	0.05
Systemic manifestations score >3	5.42	1.35–24.64	0.92	<0.001
Serum ferritin >400	4.40	1.19–19.74	0.82	<0.001

*JADAS10, 10-joint juvenile arthritis disease activity score. Non-adjusted R^2^ = 0.78, adjusted R^2^ = 0.54. AUC, the area under the receiver operating characteristic curve of the patient sample;*

* *by likelihood ratio test. OR, odds ratio; 95% CI, confidence interval*.

### Assessment of Adverse Effects

Adverse events to TCZ were reported for five patients: one developed MAS, which led to treatment discontinuation. Two patients were discontinued from treatment for moderate elevation of liver enzymes (they were also taking MTX), and hepatotoxicity resolved in both patients after withdrawal of TCZ. Moreover, two patients could not continue therapy because of severe injection site reactions.

## Discussion

Many studies reported that patients with sJIA have poorer outcome in comparison to non-systemic JIA patients. Notably, most of these studies have investigated TNFi that do not target the pro-inflammatory cytokines, typically present in sJIA (24). In our study, we investigated TCZ as first-line bDMARD in patients with sJIA after failure of csDMARDs and observed that after 1 year of treatment, 25% of the patients achieved MDA and 35% achieved CID. Furthermore, although 40% still had active disease, all these patients had lower active joints, PGA, PGE, and acute phase reactants and had no systemic manifestation at 1 year after the start of tocilizumab. Notably, we evaluated treatment outcome at 1 year to minimize the influence of initial high-dose corticosteroid administration. The rate of CID observed in our study is in the low range of the percentage of excellent response, if compared to the results reported in prior analyses of the effectiveness of IL-6 antagonists, which varies from 31 to 85% (34–36). Similar results were found in a study of 112 sJIA patients on TCZ in which 59% achieved an ACR Pedi 90 response at 1 year (20). On the other side, data from the German biologics register BIKER found a lower percentage since only 27% of the 44 patients treated with TCZ achieved an ACR Pedi 90 response at 1 year. Importantly, this study compared the effectiveness of IL-1 and IL-6 inhibitors and reported no statistical difference between the two drug cohorts (36). A recent study of the outcome of both anakinra and TCZ reported similar results to ours: 51% had achieved MDA and 39% CID of course counting those who achieved CID as primarily achieved MDA (37). The large variability of treatment response across studies may depend on differences in disease activity and severity, therapeutic protocols, timeline of response evaluation, concomitant therapies, and criteria used to assess response. Nevertheless, the varying susceptibility to IL-6 inhibition may also be explained by the pathophysiologic heterogeneity of sJIA where two distinct sJIA patient subsets based on their serum IL-6 and IL-18 levels have been postulated. The IL-6-dominant subset has a more severe polyarthritis and higher serum levels of matrix metalloproteinase, whereas the IL-18-dominant subset was more prone to develop MAS (37). This may also partially explain reduction of active joint count and JADAS-10 in our patients after tocilizumab therapy. IL-6 is a cytokine that induces fever, leukocytosis, thrombocytosis, chronic disease anemia, and increased acute phase reactants, including CRP, ESR, and serum amyloid A (37). Hence, its inhibition will result in consistent improvement in these markers. Vilaiyuk et al. (38) reported that serum IL-6 levels correlated with disease activity parameters in all sJIA patients, and this provides the etiological relation between disease activity, IL-6, and its inhibition (37). Similar results were reported in the German biologics register BIKER (35). Furthermore, among patients with active joints at treatment start, patients treated with TCZ had a higher likelihood of controlling the systemic symptoms compared to others (35). This fits with the results of our study demonstrating a significant improvement of serum ferritin levels, ESR, and CRP along with a consequent improvement of all clinical outcomes after 1-year follow-up.

We also identified that younger patients (≤7 years) with shorter disease duration (≤3 years), lower disease activity, higher serum ferritin levels, and greater systemic manifestations showed more favorable results. Similar results, except those pertaining to age, were recently reported by De Benedetti et al. (19) regarding anakinra in sJIA patients. These observations are consistent with the window of opportunity hypothesis proposing that IL-6 and IL-1 blockade may be more effective in the earlier stages of sJIA, when the disease is characterized by more prominent systemic manifestations and less severe joint disease (39). Likewise, Alexeeva et al. (40) found that earlier age, at initiation of TCZ therapy in patients with polyarticular JIA, was associated with higher chances of reaching ACR90 and pharmacological remission (40). On the other hand, the same authors reported that a higher number of swollen joints and joints with limited range of motion were associated with higher chances of remission, but in the multivariate analysis, they found that only earlier age at initiation of TCZ therapy was a statistically significant factor associated with reaching the best response to therapy in all the models (40).

It is well-known that around 40% of patients with sJIA have a monocyclic course with spontaneous remission (4, 40). Thus, the results of open studies on patients with early disease may be biased toward patients destined to a milder course. Owing to the observational nature of our study, we cannot exclude the fact that the responder group included a greater proportion of cases with more benign disease. However, our sample was composed of consecutive patients with inadequate response to csDMARDs seen in a tertiary care referral center, most of whom were resistant to or dependent on corticosteroid therapy. We acknowledge that the small sample size and the lack of a control group are the main limitations of our study. However, we believe that it adds to the knowledge on IL-6 inhibition in sJIA and may be helpful to support therapeutic decisions along with existing studies.

### Limitation Points in This Study

Almost all patients enrolled in this study were in moderate to severe disease activity, and they were taking corticosteroids as concomitant therapy, so we have to standardize the dose to avoid the effect of high doses of corticosteroids in the first phase of the disease. Secondly, various results yielded by the present study would have been more significant if a large sample had been included to overcome the effect of escaped follow-up and medication withdrawal before the study endpoint for inefficacy. So, larger studies are needed to shed additional light on this matter and ultimately improve the care of patients with sJIA.

In conclusion, we demonstrated that patients with sJIA, mainly those with shorter disease duration, younger age, and more pronounced inflammatory status, may benefit from TCZ treatment as first-line bDMARDs after failure of csDMARDs therapy. These findings may help to define the profile of patients with sJIA who are more likely to benefit from IL-6 blockade.

## Data Availability Statement

The raw data supporting the conclusions of this article will be made available by the authors, without undue reservation.

## Ethics Statement

The studies involving human participants were reviewed and approved by The local ethics committee of Tanta University Hospital with approval code 34194/8/19. Written informed consent to participate in this study was provided by the participants' legal guardian/next of kin.

## Author Contributions

All authors listed have made a substantial, direct and intellectual contribution to the work, and approved it for publication.

## Conflict of Interest

The authors declare that the research was conducted in the absence of any commercial or financial relationships that could be construed as a potential conflict of interest.
